# Cancer informatics analysis indicates high CHAC2 associated with unfavorable prognosis in breast cancer

**DOI:** 10.3389/fonc.2022.1058931

**Published:** 2022-12-09

**Authors:** Subhash Chand, Vikrant Mehta, Ratnesh K. Sharma, Anupkumar R. Anvikar, Harish Chander

**Affiliations:** ^1^ Division of Biotherapeutics, National Institute of Biologicals, Noida, India; ^2^ Department of Human Genetics and Molecular Medicine, Central University of Punjab, Bathinda, India

**Keywords:** ChaC2, breast cancer, biomarker, glutathione, prognosis, bioinformatics

## Abstract

Breast cancer remains the most commonly diagnosed cancer worldwide and exhibits a poor prognosis. The induction of genetic changes deregulates several genes that increase the disposal towards this life-threatening disease. CHAC2, a member of the glutathione degrading enzyme family has been shown to suppress gastric and colorectal cancer progression, however, the expression of CHAC2 in breast cancer has not been reported. We did an analysis of CHAC2 expression in breast cancer patients from various online tools like UALCAN, GEPIA2, GENT2, TIMER2, and bcGenExminer v4.8. Further, we used the Kaplan-Meier plotter to establish the significance of CHAC2 in BC patient survival and prognosis while TISIDB and TIMER databases were used to investigate the filtration of immune cells. The results showed that CHAC2 levels were high in breast cancer patients and elevated CHAC2 was associated with low overall survival. Taken together, the results of the present study show that like its paralog CHAC1, CHAC2 may also be an important biomarker and could have a potential therapeutic implication in breast cancer.

## Introduction

As per GLOBOCAN 2021, Breast cancer is now the most commonly diagnosed cancer with more than 2.2 million cases that resulted in close to 0.7 million deaths in the year 2020 (1).

Difficulties in early detection, resistance to conventional chemotherapy, tumor heterogeneity, and the associated side-effects of the treatment are a few among many hurdles that lead to worse prognosis and overall survival (OS) of breast cancer patients thus warranting identification of new biomarkers for early cancer detection and development of novel treatment strategies (2–4). Identifying novel biomarkers responsible for tumor progression may provide a better course of treatment, and may also contribute in improving the prognosis of breast cancer patients (5, 6) Located on chromosome no 2 (2p16.2), Cation transport regulator homolog 2 (CHAC2) is one of the members of the CHAC family that catalyzes the glutathione degradation to 5-oxoproline and cystein-glycine, however with lower catalytic efficiency compared to CHAC1 (7). Cation transport regulator homolog 1 (CHAC1), the other member of the CHAC family was discovered as a proapoptotic component of the unfolded protein response (UPR) pathway (8). The aberrant expression of CHAC1 surprisingly has been linked with both tumor suppression as well as tumor progression (9–13). Liu et al. (14) reported that CHAC2 essentially acted as a tumor suppressor in gastric and colorectal cancer as evident from *in vitro* and *in vivo* studies.

We have recently shown that high levels of CHAC1 are associated with increased proliferation, lymph node metastasis, and poor prognosis in breast cancer (15, 16). However, the expression of CHAC2 in breast cancer and its association with the prognosis of breast cancer is unknown. In the present study, we have investigated the transcriptomic data of CHAC2 expression in breast normal-like vs. tumor samples and molecular subtypes. Further online databases (UALCAN, UCSCxena, Enrichr, GEPIA2, and GeneMANIA) deciphered CHAC2 expression in breast cancer through gene ontology studies, interaction, and correlation analysis. Kaplan-Meier plotter analysis established the prognostic signifcance of CHAC2 expression in breast cancer.

## Material and methods

### Analysis of CHAC2 gene expression in Breast Cancer according to various clinicopathological parameters

Differential analysis of CHAC2 gene expression in breast vs normal tissues was carried out using UALCAN web server (http://ualcan.path.uab.edu) (17), GEPIA2 (http://gepia2.cancer-pku.cn) (18), bc-GenExMiner v4.8 (http://bcgenex.ico.unicancer.fr) (19). A pan-cancer analysis of CHAC2 expression was done with the help of UALCAN, GENT2 (http://gent2.appex.kr/gent2/) (20), TNMplot (https://tnmplot.com/analysis/) (21), and TIMER2.0 (http://timer.cistrome.org/) (22) databases. Further, we utilized UALCAN, bc-GenExMiner v4.8, and GEPIA2 to analyse the *CHAC2* gene expression on the basis of various clinicopathological parameters such as molecular subtypes, tumor stages, lymph node metastasis status, age, Nottingham Prognostic index (NPI), and Scarff-Bloom-Richardson (SBR) grading.

### CHAC2 gene expression according to promoter methylation status and gene mutation

The promoter methylation and CHAC2 expression was analysed with UALCAN database according to normal vs tumor samples followed by normal vs molecular subtypes of breast cancer. The analysis of CHAC2 expression with respect to p53 mutation was carried out with the help of UALCAN and bc-GenExMiner v4.8. We further utilized muTarget database which performs the data processing in R statistical environment and uses the TCGA database to acquire RNA-sequencing and mutation data. The database contains 7876 solid tumor samples from 18 diverse tumor types with both somatic mutation and RNA-seq data. Running parameters used to do the analysis were FC>1.4, *p* < 0.01, and at least 2% mutation prevalence (23).

### Survival analysis and prognostic significance of CHAC2 expression in breast cancer

Analysis of the survival data was executed with Kaplan-Meier plotter (https://kmplot.com/analysis/) which is a widely used tool to evaluate the correlation of genes of interest with survival times based on mRNA, miRNA, or protein expression in more than 30000 samples from 21 tumor types (24). We analyzed the CHAC2 expression (235117_at) in terms of disease mean-free survival (DMFS), overall survival (OS), and relapse-free survival (RFS) from Kaplan-Meier plotter. Further, we used bc-GenExMiner v4.8 and GENT2 database to determine the survival difference between low and high CHAC2 expression groups.

### Immune cell infiltration analysis

TIMER database is used to investigate the tumor-immune interaction (http://timer.cistrome.org/). TIMER database provides the infiltration of six different immune infiltrates like B cell, CD4+ T cell, CD8+ T cell, neutrophil, macrophage, and dendritic cells. TIMER database was further used to estimate whether the expression of CHAC2 correlated with immune cell infiltration (25). Moreover, it also provides gene expression data, clinical outcomes, somatic mutations, and somatic copy number alterations in various cancers (25). Wilcoxon test is used to calculate the statistical significance of data obtained from this database. Besides TIMER, we also used the TISIDB database (http://cis.hku.hk/TISIDB/index.php) to elucidate the infiltration of various immune cells with respect to CHAC2 expression in Pan-cancer analysis and later with Breast Invasive carcinoma (26).

### Gene correlation studies on CHAC2

We obtained top 10 most closely correlated genes from GEPIA2 database and obtained a heat map depicting this correlation with the help of bc-GenExMiner v4.8. Alternatively, the correlation graph of CHAC2 with the most closely correlated gene was generated using UALCAN, GEPIA2, and UCSC Xena database. UCSC Xena (https://xena.ucsc.edu/) can be used to explore multi-omics data from large public TCGA and GDC genomics datasets (27). The expression data of a particular gene can be visualized in terms of expression, DNA methylation, phenotypic annotations, and ATAC-seq signals.

### Gene ontology studies and enrichment analysis

The gene ontology (GO) studies of CHAC2 were done using the Enrichr server (https://maayanlab.cloud/Enrichr/). The signaling pathways and the functional enrichment of co-expressed genes were categorized into biological processes, molecular functions, and cellular components (28, 29).

### Gene interaction network analysis

GeneMANIA database (http://genemania.org) was used to plot the gene interaction network of CHAC2 (30). Maximum resultant genes were set to ‘20’ and maximum resultant attributes were set to ‘10’. Further top 10 correlated genes as obtained from the GEPIA2 database were uploaded as a gene set and another interaction network was obtained.

## Results

### CHAC2 expression is high in breast tumors compared to adjacent normal tissues

**Figure 1:**
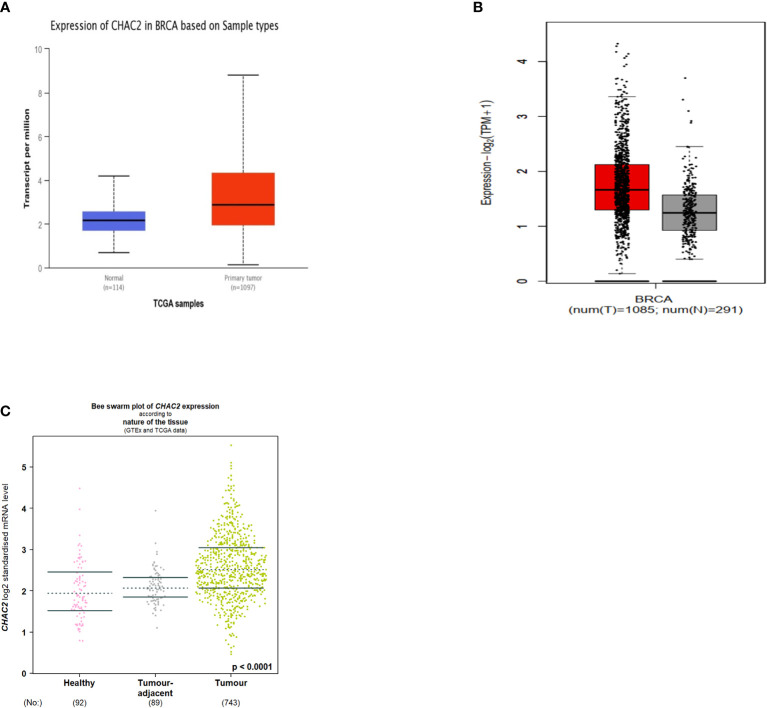
CHAC2 transcript levels in cancer and normal human breast tissues according to **(A)** UALCAN (Box Plot), **(B)** GEPIA2 (Box Plot), **(C)** bcGenExminer v4.8 (Bee swarm plot).

The different databases to demonstrate the CHAC2 expression in breast cancer. CHAC2 was expressed at high levels in tumor tissues compared to adjacent normal tissues as per UALCAN (**Figure 1A**) and the GEPIA2 database (**Figure 1B**). We further looked into the CHAC2 expression patterns using the bcGenexminer database within the adjacent tumor area and the transcript levels of CHAC2 were high in tumor samples compared to adjacent normal and healthy controls (**Figure 1C**). Subsequently, a pan-cancer analysis of CHAC2 expression in various cancers and we attempted the same by utilizing UALCAN (**Figure S1A**), TNMplot (**Figure S1B**), GENT2 (**Figure S1C**), and TIMER2 (**Figure S1D**) database to perform the analysis. In summary, we found significantly high CHAC2 expression (*p*-value <0.0001) in breast tumor cases compared to their normal counterparts (**Figure 1**).

### CHAC2 expression and its correlation with breast cancer subtypes, stage, and node metastasis

**Figure 2:**
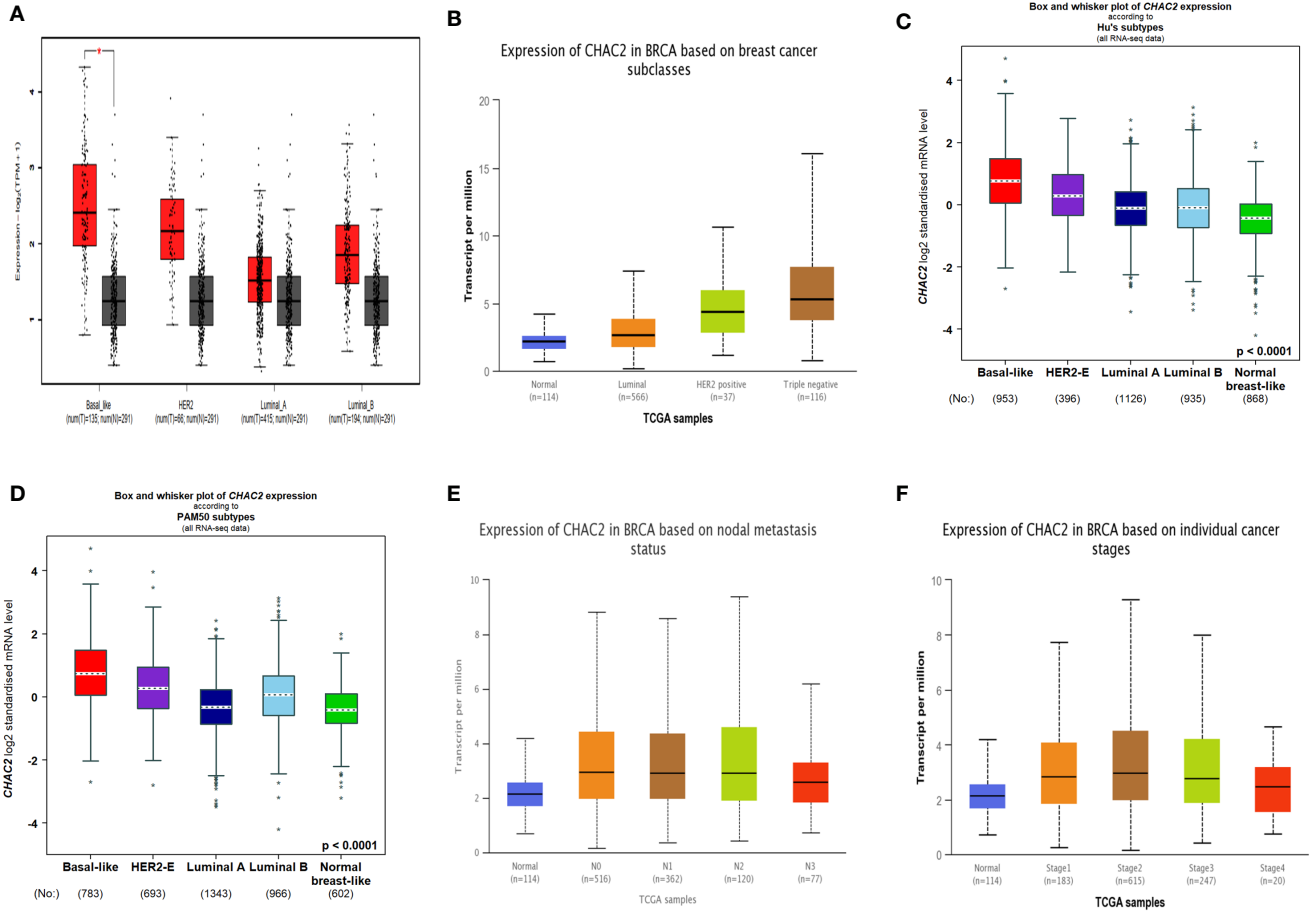
Molecular subtypes of breast cancer and CHAC2 expression according to **(A)** GEPIA2, **(B)** UALCAN, **(C, D)** bcGenExminer v4.8 **(E)** CHAC2 expression and its association with the status of node metastasis according to UALCAN **(F)** CHAC2 expression and its association with the breast cancer stage according to UALCAN analysis. *p < 0.05.

Breast cancer can be divided into four molecular subtypes based on the expression of certain markers like estrogen receptor (ER), progesterone receptor (PR), and Human epidermal growth factor receptor 2 (HER2) while the subtype lacking the expression of all these receptors is known as triple-negative breast cancer (TNBC) (31). Importantly, HER2 and TNBC are associated with poor survival and frequent relapse (31). Our analysis found that CHAC2 expression was significantly high (*p*-value<0.0001) in these aggressive subtypes compared to luminal (A & B) subtypes from multiple databases (**Figures 2A-D**). We further investigated if high CHAC2 correlated with the stage and positive node metastasis of the tumor. Unsurprisingly, the overall CHAC2 expression enhanced with the node metastasis (**Figure 2E**) and stage (**Figure 2F**) of the breast tumor indicating that high CHAC2 expression correlated with increasing tumor stage and node metastasis.

### CHAC2 expression and its correlation with clinicopathologic parameters of breast cancer

To evaluate the CHAC2 expression according to various clinicopathologic parameters, bcGenexminer v4.8 was used. The analysis was done based on various parameters as indicated (**Table 1**). In summary, CHAC2 mRNA expression was significantly enhanced (*p*-value=0.0004) in women with age ≤ 51. Similarly, tumors with ER- status, PR-, Her2+, Basal-like, and TNBC had significantly high CHAC2 transcript levels (*p*-value<0.0001). Moreover, the analysis according to the prognostic index like Nottingham prognostic index (NPI) and Scarff Bloom and Richardson (SBR) grade status revealed a direct correlation of CHAC2 expression with increasing tumor grade and stage (**Table 1**).

**Table 1:** The relationship between CHAC2 mRNA expression and clinicopathological features of breast cancer from bc-GenExMiner v4.8 database.

Characteristics	Number	mRNA	*p*-value
**Age (years)**			
≤51	1021	↑	0.0004
>51	2995		
**ER**			
–	510	↑	<0.0001
+	3685		
**PR**			
–	746	↑	<0.0001
+	3312		
**HER2**			
–	3446		
+	615	↑	<0.0001
**ER/PR combinations**			
ER+/PR+	3262		
ER+/PR-	283		
ER-/PR+	45		
ER-/PR-	462	↑	<0.0001
**Basal-like and Triple Negative status**			
Non-basal-like and non-TNBC	3479		
Basal-like and TNBC	246	↑	<0.0001
**NPI status**			
NPI1	1173		
NPI2	1525		
NPI3	416	↑	<0.0001
**SBR status**			
SBR1	544		
SBR2	1699		
SBR3	1374	↑	<0.0001

↑, upregulated expression. In the case of ER/PR combinations, the ↑ indicates the highest expression in this group. ER,Estrogen receptor; PR,Progesterone receptor; HER2, Human epidermal growth factor receptor 2; NPI, Nottingham Prognostic Index; SBR, Scarff Bloom and Richardson grade status.

### High CHAC2 expression correlates with mutant p53 expression and may be due to methylation changes within CHAC2 promoter

**Figure 3:**
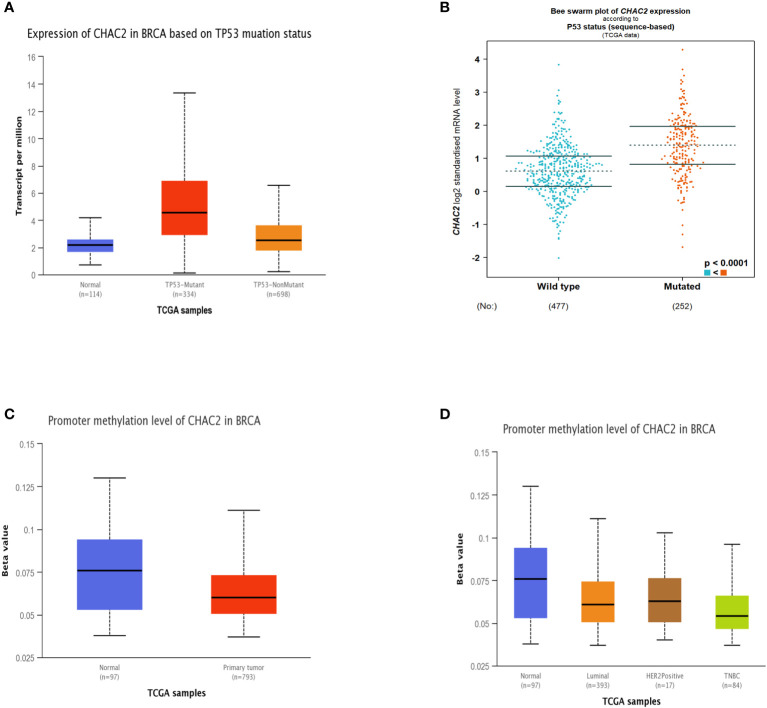
Impact of p53 mutation and promoter methylation on CHAC2 expression in breast cancer patients and normal samples. CHAC2 levels in mutant p53 vs non-mutant or wild-type p53 patients as per **(A)** UALCAN, **(B)** bcGenExminer v4.8. Promoter methylation of CHAC2 and its expression from UALCAN database as per **(C)** normal vs tumor samples and **(D)** normal vs molecular subtypes of breast cancer.

Since mutant p53 induces the expression of several genes that enhances carcinogenesis (32–34), we extended our analysis to see if CHAC2 levels were enhanced in the “p53 mutant” group compared to the “p53 non-mutant” or “wild-type” group. The analysis showed that CHAC2 levels were high in the former category as evidenced by UALCAN (**Figure 3A**) and bcGenexminer (**Figure 3B**) (*p*-value<0.0001) databases.

Additionally, we analyzed the promoter methylation levels of *CHAC2* as changes in the methylation status of various oncogenes and tumor suppressors are linked with cancer progression (35). Our analysis showed that the *CHAC2* promoter was significantly hypomethylated in breast tumor cases (**Figure 3C**). Moreover, the analysis of methylation in terms of breast cancer subtypes showed that among all the molecular subtypes, TNBC had the most down-regulated methylation levels (**Figure 3D**).

### CHAC2 expression may correlate with mutations in crucial genes related to breast cancer

Since we found that expression of CHAC2 enhanced with p53 mutation, therefore, we extended our analysis to see if the CHAC2 levels correlated with the mutations in other key genes. muTarget tool was used to identify these genes. The top ten most strongly associated genes with CHAC2 expression have been described (**Table 2**) while expression of CHAC2 with wild-type or mutant forms of indicated genes have been shown (**Table 2**). As results indicate, mutations in the TP53 are most prevalent (mutation rate: 34.3%) followed by CDH1 (mutation rate: 14.1%), BIRC6 (mutation rate: 3.47%), DYNC2H1 (mutation rate: 3.26%), and UTRN (mutation rate: 2.96%). These findings suggest that CHAC2 expression is closely linked to gene mutations in breast cancer.

**Table 2:** The top ten mutant genes most closely associated with CHAC2 expression and their features. (FC, Fold change).

Mutated gene	Mean expression (mutant)	Mean expression (wild)	No. of mutant	No. of wild	FC (mut/wild)	Direction	*p-*value
TP53	105.42	58.13	336	643	1.81	Up	1.62e-34
CDH1	51.99	78.03	138	841	1.49	Down	3.94e-08
HRNR	117.95	73.45	20	959	1.61	Up	2.21e-04
DYNC2H1	113.81	73.03	32	947	1.56	Up	3.41e-04
UTRN	109	73.3	29	950	1.49	Up	4.63e-04
BRCA2	111.58	73.42	24	955	1.52	Up	5.63e-04
PAPPA2	124.3	73.32	20	959	1.7	Up	9.47e-04
OTOGL	108.29	73.61	21	958	1.47	Up	1.16e-03
BIRC6	107.12	73.18	34	945	1.46	Up	1.16e-03
ATRX	111.07	73.32	27	952	1.51	Up	1.19e-03

### High CHAC2 expression leads to poor prognosis and overall survival

**Figure 4:**
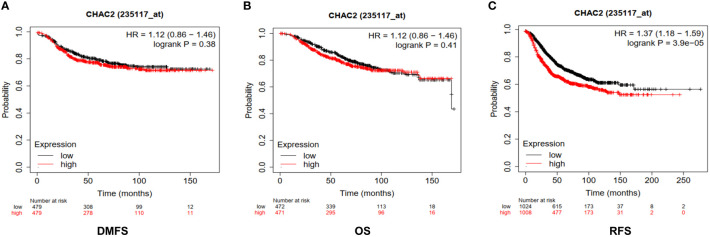
Prognostic significance of CHAC2 in the survival of breast cancer patients. Kaplan-Meier survival plots were obtained from Km plotter indicating lower survival of breast cancer patients in terms of **(A)** DMFS, **(B)** OS, and **(C)** RFS. DMFS, Distant metastasis-free survival; OS, Overall Survival; RFS, Relapse-free survival.

We found high expression of CHAC2 in breast tumor samples and in aggressive subtypes like HER and TNBC compared to luminal subtypes. Therefore, we examined if high CHAC2 levels correlated with survival probability and prognosis with the help of a Kaplan-Meier plotter. The survival plots obtained from Km plotter (**Figures 4A-C**) indicated that high CHAC2 expression correlated significantly with shorter survival indicating that elevated CHAC2 expression negatively associated with the prognosis of breast cancer patients (**Figures 4A-C**).

### Association between CHAC2 and immune cell filtration

**Figure 5:**
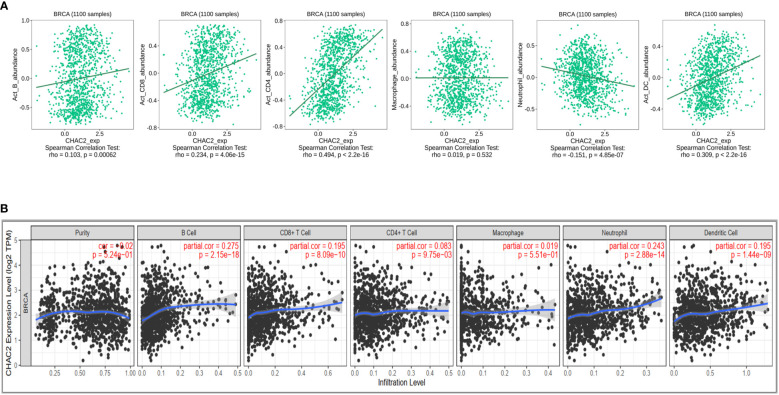
Correlation of CHAC2 expression and immune filtration in BC. **(A)** Spearman correlation from TISIDB between CHAC2 and B cells, CD4+ T cells, CD8+ T cells, macrophage, neutrophil, and dendritic cells. **(B)** Analysis of correlation between CHAC2 and immune cells from TIMER database.

Immune cells constitute an important component of the tumor microenvironment that has been implicated in facilitating breast cancer progression and invasion (36). Moreover, immune cells have been reported to be an independent predictor of patient survival and response to drug therapy (37, 38). We initially did a Pan-cancer analysis of CHAC2 expression with immune cell infiltration using TISIDB (**Supplementary Figure S2**). We then selected “Breast Invasive Carcinoma” and analyzed the correlation of various immune cells with CHAC2 expression (**Figure 5A**). We found a significant positive correlation of activated B cells (Spearman: rho=0.103, *p*=0.00062), CD4+ (Spearman: rho=0.494, *p*<2.2e-16) and CD8+ T cells (Spearman: rho=0.234, *p*=4.06e-15), and dendritic cells (Spearman: rho=0.309, *p*<2.2e-16). Conversely, a negative correlation was observed with neutrophil infiltration (Spearman: rho=-0.151, *p*=4.85e-07) while a weakly positive and non-significant correlation of macrophages was observed (Spearman: rho=0.019, *p*=0.532). Additionally, we used the TIMER database to corroborate the immune filtration analysis (**Figure 5B**). The result of immune filtration analysis shows that CHAC2 expression may be involved in the regulation of immune cells in breast cancer.

### Correlation analysis of genes linked to CHAC2 in BC

**Figure 6:**
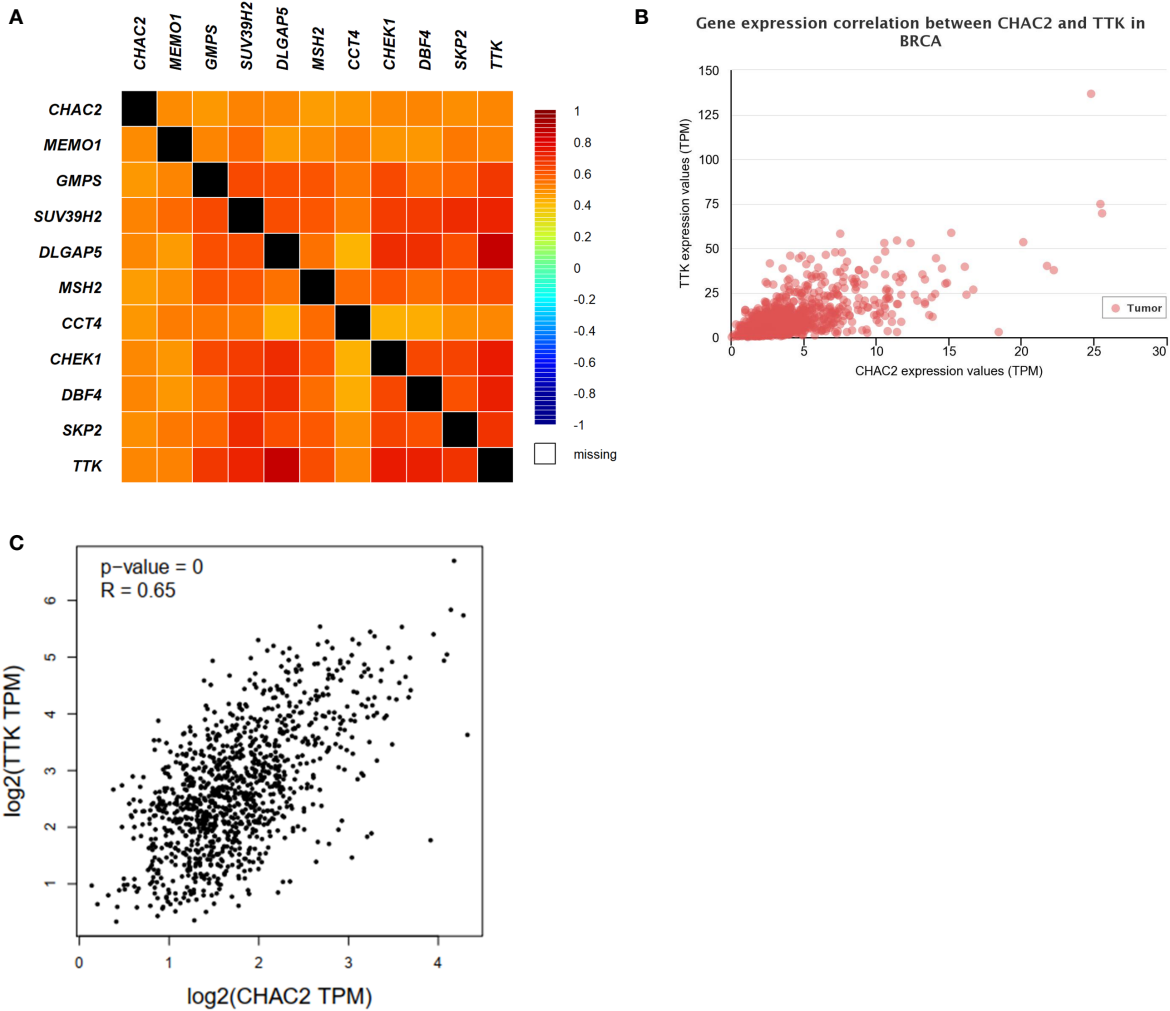
Analysis of CHAC1 correlations and top 10 correlated genes. **(A)** Heat map depicting the CHAC2 correlation with top 10 closely related genes. Correlation between CHAC2 and TTK from **(B)** UALCAN, **(C)** GEPIA2.

The top 10 most closely correlated genes with *CHAC2* were obtained from GEPIA2 using the TCGA dataset. The heat map was then generated using bcGenExMiner v4.8 (**Figure 6A**). Among all, the most closely correlated gene was TTK as obtained from UALCAN (Pearson correlation coefficient = 0.66) (**Figure 6B**), and GEPIA2 (Pearson correlation coefficient = 0.65) (**Figure 6C**). TTK/hMPS1 gene is known to play a role in centrosome duplication, and mitotic checkpoint signaling and is known to play a role in the progression of breast cancer (39, 40). Further, we did a correlation analysis of CHAC2 with the TTK gene using UCSCxena (**Figure S3A**). TIMER database was used to obtain the plots of top 10 closely related genes (**Figure S3B**).

### Gene ontology (GO) studies and interaction networks related to CHAC2

We chose the top 100 related genes that expressed with CHAC2 through the Enrichr database. To further understand the gene ontological features and signaling pathways related to the CHAC2 gene, we did enrichment analysis from various gene set libraries including GO biological process (**Figure S4A**), GO molecular function (**Figure S4B**), GO cellular component (**Figure S4C**), KEGG 2021 (**Figure S4D**), and Bioplanet 2019 (**Figure S4E**). The results of cluster analysis showed that CHAC2 and its correlated genes are significantly enriched in various pathways and cellular processes associated with DNA replication, damaged DNA binding, cell cycle, oocyte meiosis, etc.

We constructed a protein-protein interaction (PPI) network for CHAC2 and its 20 related genes as obtained from the GeneMANIA database (**Figure S4F**). These genes included CHAC1, UBL5, DPYD, DPH5, ATP13A3, AC005154.5, GGACT, GGCT, POLR3A, CDKN2AIP, HAPLN3, SH3GLB1, CRK, FSD1, SAP18, UBA1, TIPIN, CNBP, DEPDC1B, and DHX29. Another PPI network for the top 10 correlated genes of CHAC2 as obtained previously from GEPIA2 was constructed (**Figure S4G**).

## Discussion

Breast cancer is a heterogeneous disease that progresses due to various factors including genetic mutations, over-expression of various receptors, cytoskeletal rearrangement, and epithelial-mesenchymal transition that ultimately transcends into metastatic disease (41–43). Owing to its high incidence and mortality rate, early detection of breast cancer, advancement in precision medicine, and identification of new biomarkers are highly necessary. Both the members of the CHAC family are UPR genes and have been implicated in cancer progression (14, 16). While several studies have reported a dual role for CHAC1 in cancer progression, studies depicting the association of CHAC2 in carcinogenesis are sparse. To the best of our knowledge, this is the first study that reports the expression of CHAC2 in breast cancer. Previously, CHAC2 has been shown to behave as a tumor suppressor in gastric cancer cells transfected with CHAC2 led to enhanced ROS levels, mitochondrial apoptosis, and autophagy through the UPR pathway (14).

From the pan-cancer analysis it is clear that CHAC2 has a different expression in different tumor tissue and therefore, mechanistic studies are warranted to conclude the factors behind the differential expression. Based on the results of the present study, we showed that CHAC2 is highly expressed in breast cancer and seems to be associated with node metastasis, and stage of breast cancer through various databases. We found that CHAC2 mRNA expression enhanced with increasing tumor grading according to NPI and SBR grading (44). Moreover, CHAC2 over-expression in the HER2 and TNBC is indicative of the fact that high CHAC2 may serve as a biomarker for these aggressive subtypes. Another member of the CHAC family, CHAC1 has been previously reported to be highly expressed in these subtypes, and on similar lines, CHAC2 also showed a prognostic significance as per survival curves obtained from the Kaplan-Meier plotter (15).

p53 also known as the “guardian of the genome” is mutated in more than 50% of human tumors (34). Due to its highly important role in cell cycle regulation, mutations in p53 seem to be a prerequisite for tumors with high grade, invasion, and metastatic capability (45, 46). Mutations in key genes such as p53 have been highly implicated in various human malignancies and its mutations have been reported to escalate carcinogenesis (34, 47). From various databases like UALCAN, bcGenexminer, and muTarget, the analysis showed that tumor samples with mutant p53 showed high CHAC2 expression. Moreover, epigenetic changes such as DNA methylation have been linked with progression of cancer including breast tumorigenesis (35, 48). Our analysis showed that hypo methylation of CHAC2 was prevalent in tumor samples. Furthermore, the analysis of gene mutations associated with CHAC2 using muTarget revealed important genes associated with CHAC2 expression. Among them, the most significant genes turned out to be *tp53* which encodes p53, and *CDH1* which encodes E-cadherin. The loss of E-cadherin enhances the epithelial-mesenchymal transition (EMT), lymph node metastasis, and associates with poor differentiation of breast cancer (49, 50). The present work found low expression of CHAC2 in CDH1 mutant samples while high CHAC2 expression was observed in the samples with mutations in genes such as HRNR, UTRN, and DYNC2H1. This paves a way for future therapies targeting CHAC2 in breast cancer.

With the advent of immunotherapy-like treatments in the last decade for cancer, it is now clear that immune response toward cancer cells has important implications for disease progression and response to therapy. Thus, we investigated the correlation between CHAC2 and immune cell infiltration from different databases and found that a positive correlation of CHAC2 existed with the infiltration of activated CD8+, CD4+ T cells, and activated dendritic cells indicating that CHAC2 may have an important role to play in the infiltration of these immune cells and ultimately of the breast tumor microenvironment. Apart from this, the correlation analysis revealed a strong correlation of CHAC2 with TTK which is a tyrosine kinase. In the past, several reports have suggested an oncogenic role for TTK in breast cancer (40, 51). For example, TTK regulates the EMT in triple-negative breast cancer cells and its knockdown led to reduced viability and colony formation ability in MDA-MB-231 and Hs578t cells (51). Moreover, Morrison et al. (52) showed that TTK knockdown in MDA-MB-231 cells correlated with reduced organoid growth in 3-D cultures. From the correlation analysis, it can therefore be inferred that both CHAC2 and TTK may be potential candidate for future therapeutic targets in breast cancer. The gene function and enrichment studies showed that CHAC2 and its correlated genes may be involved in DNA replication, DNA repair, cell cycle, RNA binding, damaged DNA binding, oocyte meiosis and maturation. Taken together, our study explored the CHAC2 expression in breast cancer datasets and aimed to establish its clinical significance in breast cancer. Therefore, considering the totality of the evidence reported in this study, it is clear that high CHAC2 expression associates with more progressive disease suggesting a novel therapeutic target for breast cancer as well as a biomarker for the aggressiveness of the breast tumor. Our study warrants further preclinical research with various *in vitro* and *in vivo* models to ascertain the molecular mechanisms that regulates CHAC2 expression which may be beneficial in breast cancer diagnosis and treatment.

## Data availability statement

The original contributions presented in the study are included in the article/**Supplementary Material**. Further inquiries can be directed to the corresponding author.

## Author contributions

SC: Analysed the data, prepared figures in part, and wrote the manuscript. VM: Analysed the data, designed the study, prepared figures, and wrote the manuscript. RS: Designed the study in part. AA: Design and analysis of the study in part, manuscript editing. HC: Design of study, funding acquisition, review, and editing of the manuscript. All authors contributed to the article and approved the submitted version.

## Supplementary material

Supplementary Figure 1 Pan-cancer analysis of CHAC2 gene expression according to **(A)** UALCAN, **(B)** TNMplot, **(C)** GENT2, and **(D)** TIMER2.0. Breast cancer has been indicated in a red rectangular box.

Supplementary Figure 2 Heat map depicting the correlation of CHAC2 with 28 different immune cells. Red color indicates more positive while blue color indicated more negative correlation.

Supplementary Figure 3
**(A)** Heat map of CHAC2 and TTK mRNA expression using UCSCXena database. **(B)** Correlation of CHAC2 and top 10 correlated genes from TIMER database.

Supplementary Figure 4 Protein-protein interaction network and Gene ontology study of CHAC2. Gene ontology (GO) from Enrichr database according to **(A)** GO biological process 2021, **(B)** GO molecular function 2021, **(C)** GO Cellular component 2021, **(D)** KEGG 2021 Human, and **(E)** Bioplanet 2019. The length and intensity of the color of the bar indicate the level of significance (a more intense color indicates high significance). **(F)** Protein-protein interaction network of CHAC2 and its top 20 interactive genes was obtained from GeneMANIA. **(G)** Protein-protein interaction network of top 10 CHAC2 correlated genes.

Supplementary Figure 5 Schematic outline of the methodology for cancer informatics analysis of CHAC2 expression.
