# Supplementary figures and images for: Cancer informatics analysis indicates high CHAC2 associated with unfavorable prognosis in breast cancer
Source: Front Oncol. 2022 Dec 9;12:1058931. doi: 10.3389/fonc.2022.1058931 (PMC9780439; doi:10.3389/fonc.2022.1058931)

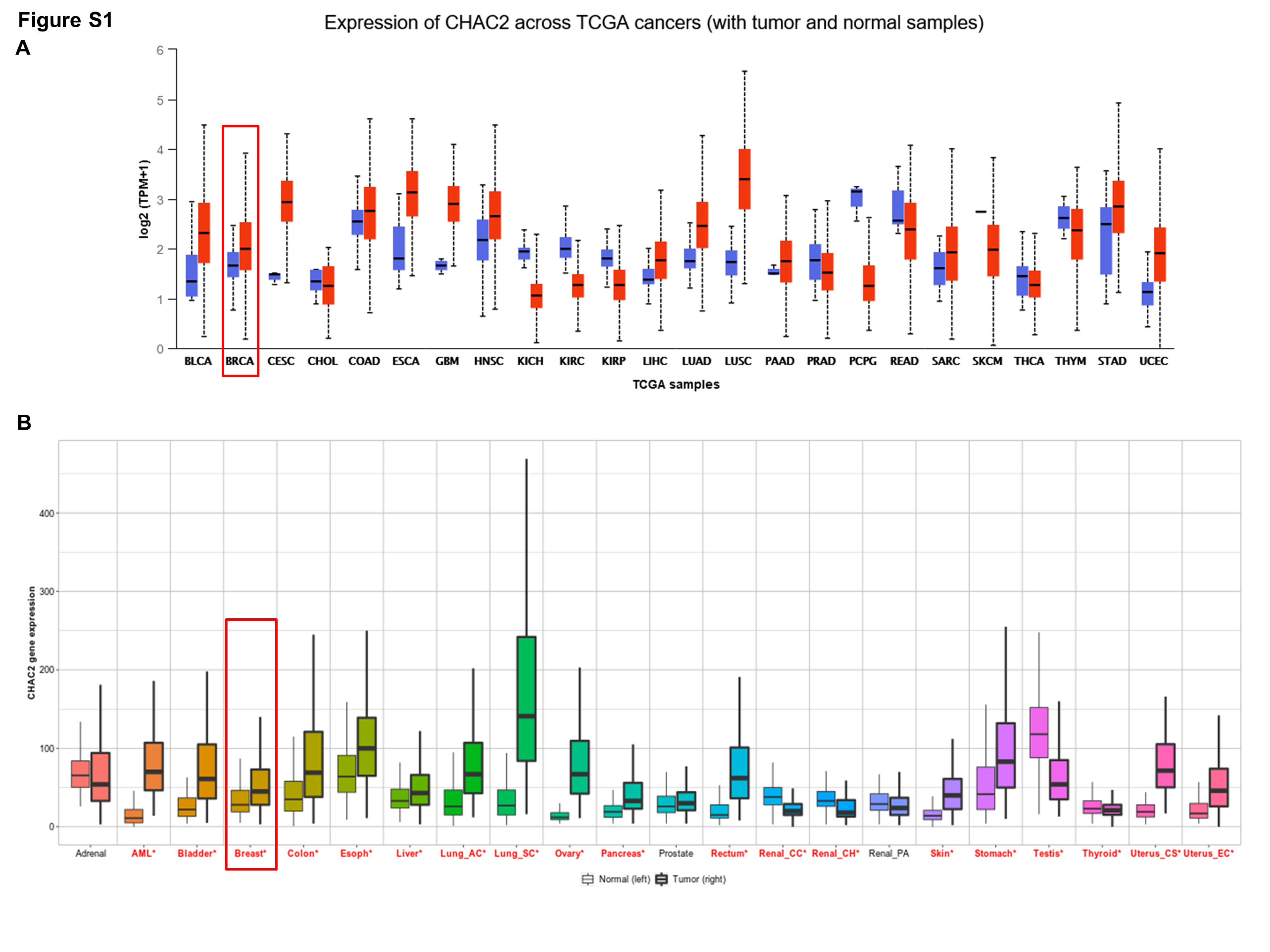

Supplement: Supplementary Figure 1 — Pan-cancer analysis of CHAC2 gene expression according to (A) UALCAN, (B) TNMplot, (C) GENT2, and (D) TIMER2.0. Breast cancer has been indicated in a red rectangular box. [file Image_1.tif]

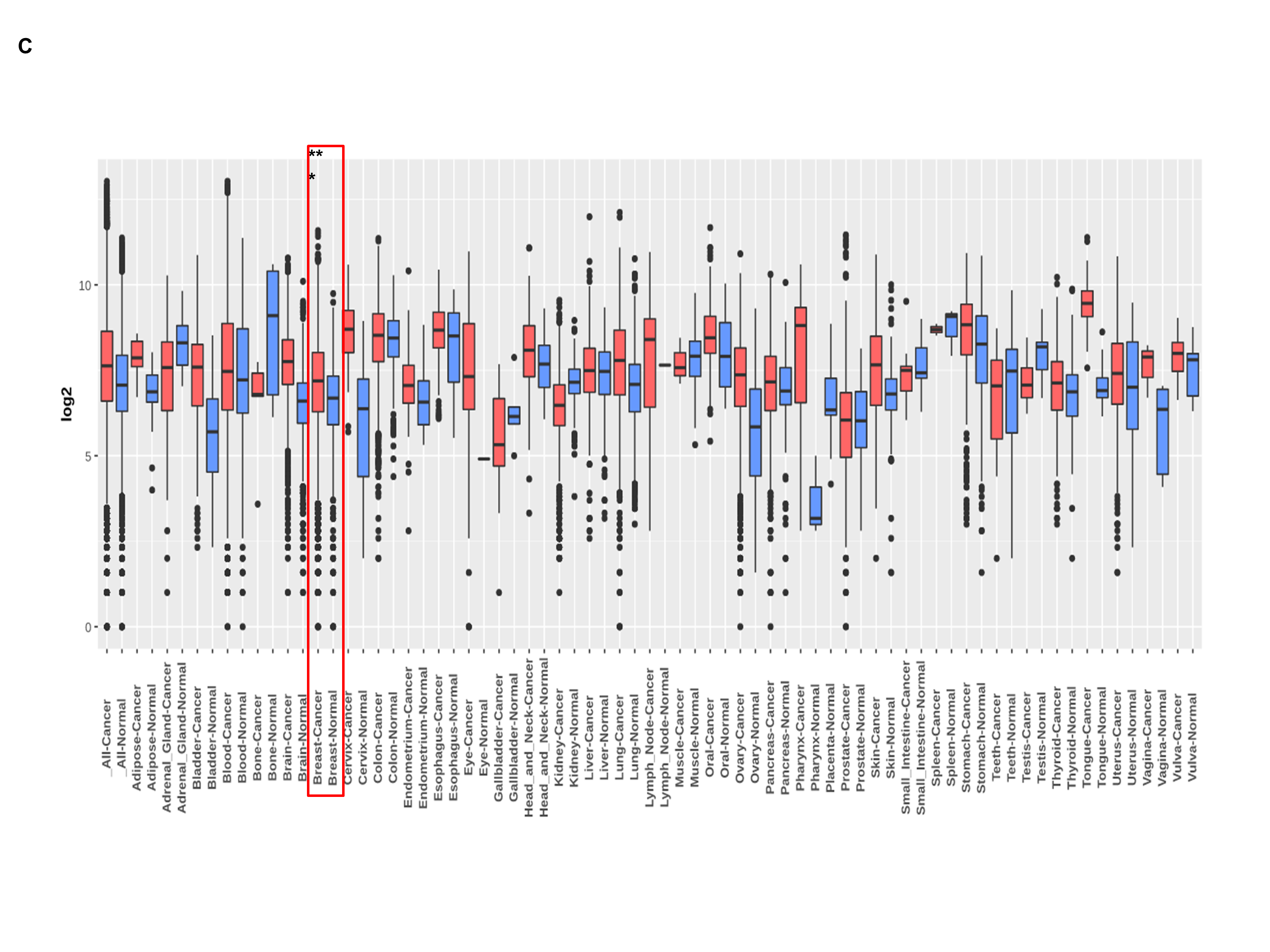

Supplement: Supplementary Figure 2 — Heat map depicting the correlation of CHAC2 with 28 different immune cells. Red color indicates more positive while blue color indicated more negative correlation. [file Image_2.tif]

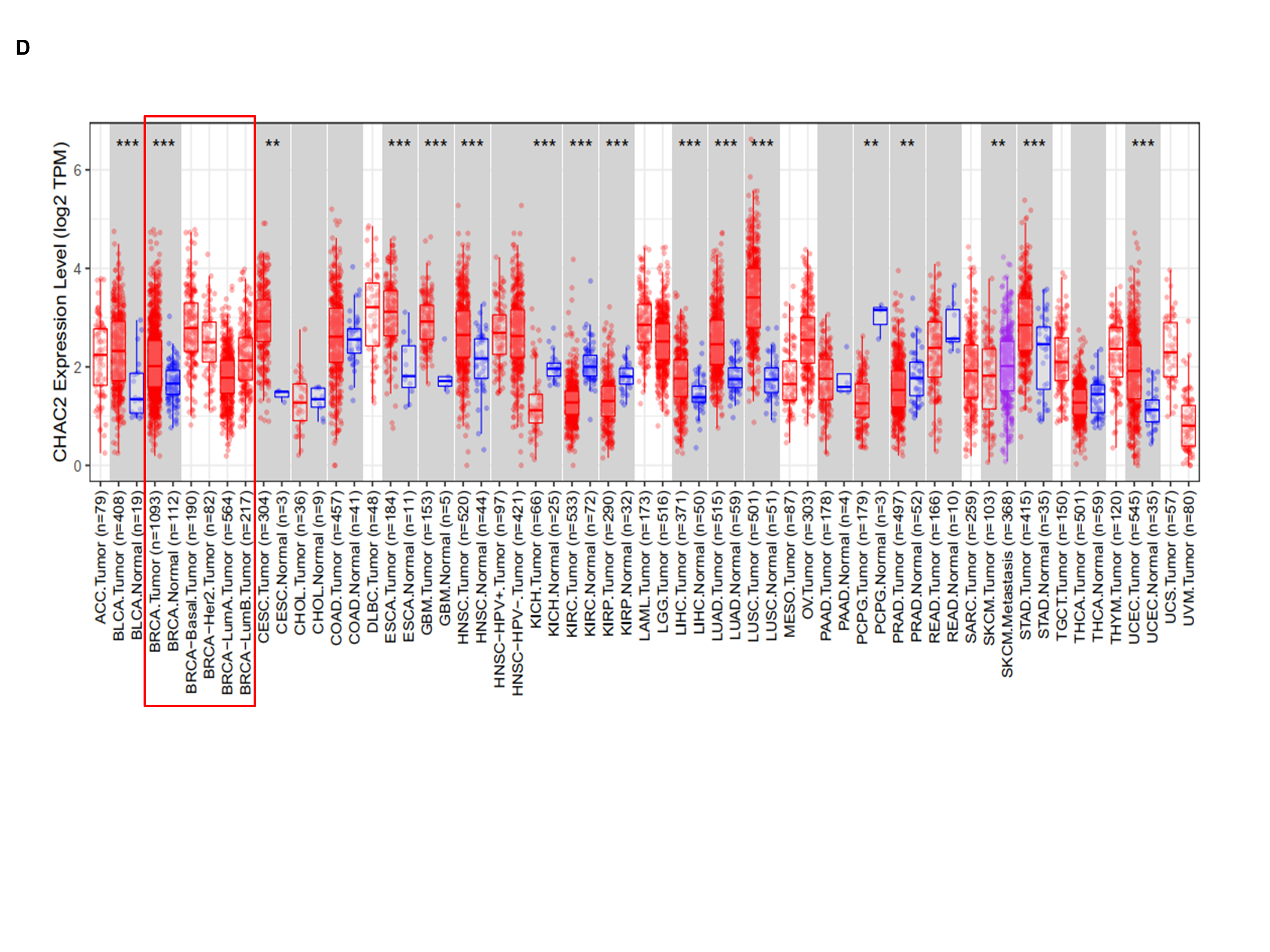

Supplement: Supplementary Figure 3 — (A) Heat map of CHAC2 and TTK mRNA expression using UCSCXena database. (B) Correlation of CHAC2 and top 10 correlated genes from TIMER database. [file Image_3.tif]

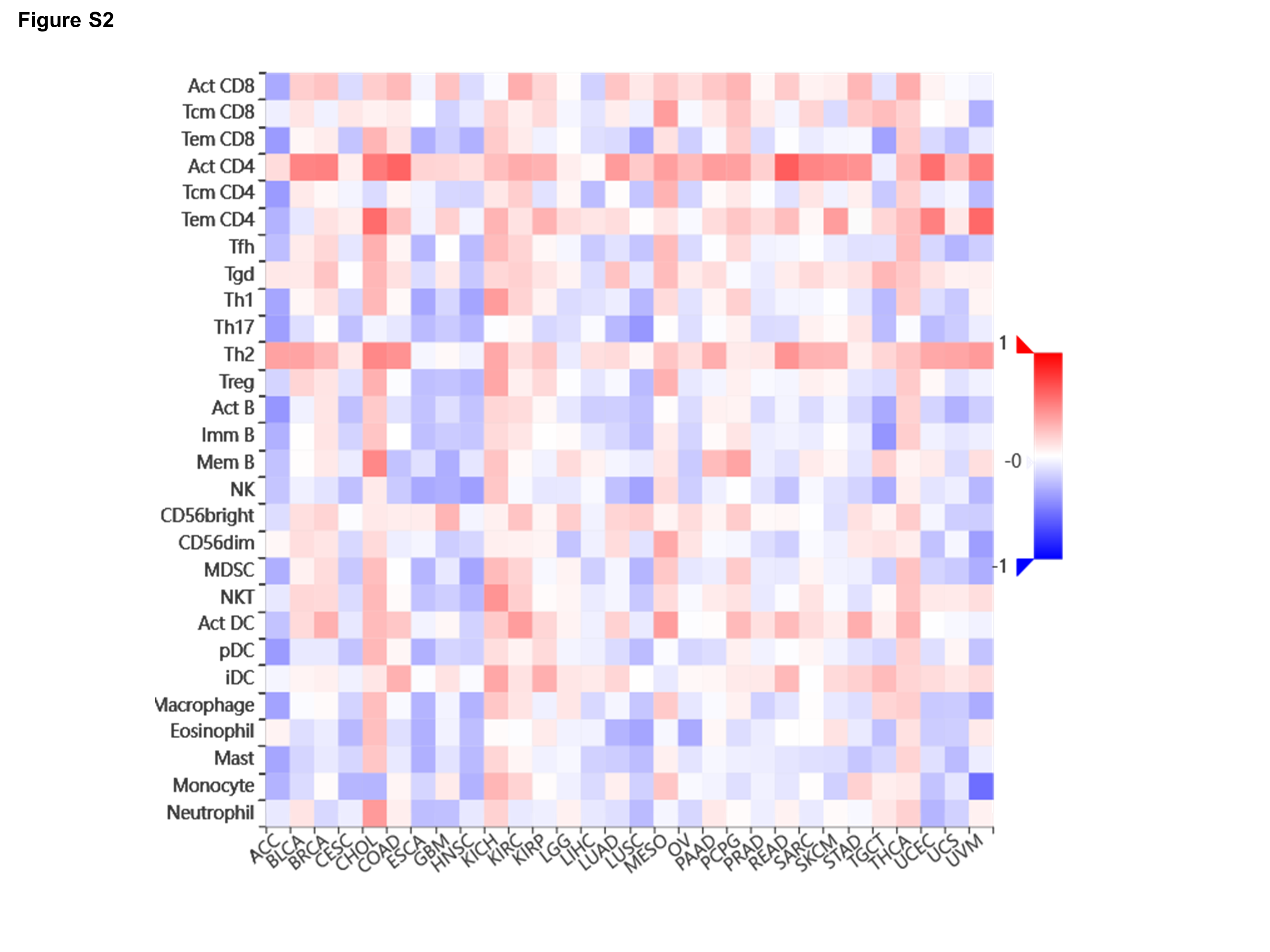

Supplement: Supplementary Figure 4 — Protein-protein interaction network and Gene ontology study of CHAC2. Gene ontology (GO) from Enrichr database according to (A) GO biological process 2021, (B) GO molecular function 2021, (C) GO Cellular component 2021, (D) KEGG 2021 Human, and (E) Bioplanet 2019. The length and intensity of the color of the bar indicate the level of significance (a more intense color indicates high significance). (F) Protein-protein interaction network of CHAC2 and its top 20 interactive genes was obtained from GeneMANIA. (G) Protein-protein interaction network of top 10 CHAC2 correlated genes. [file Image_4.tif]

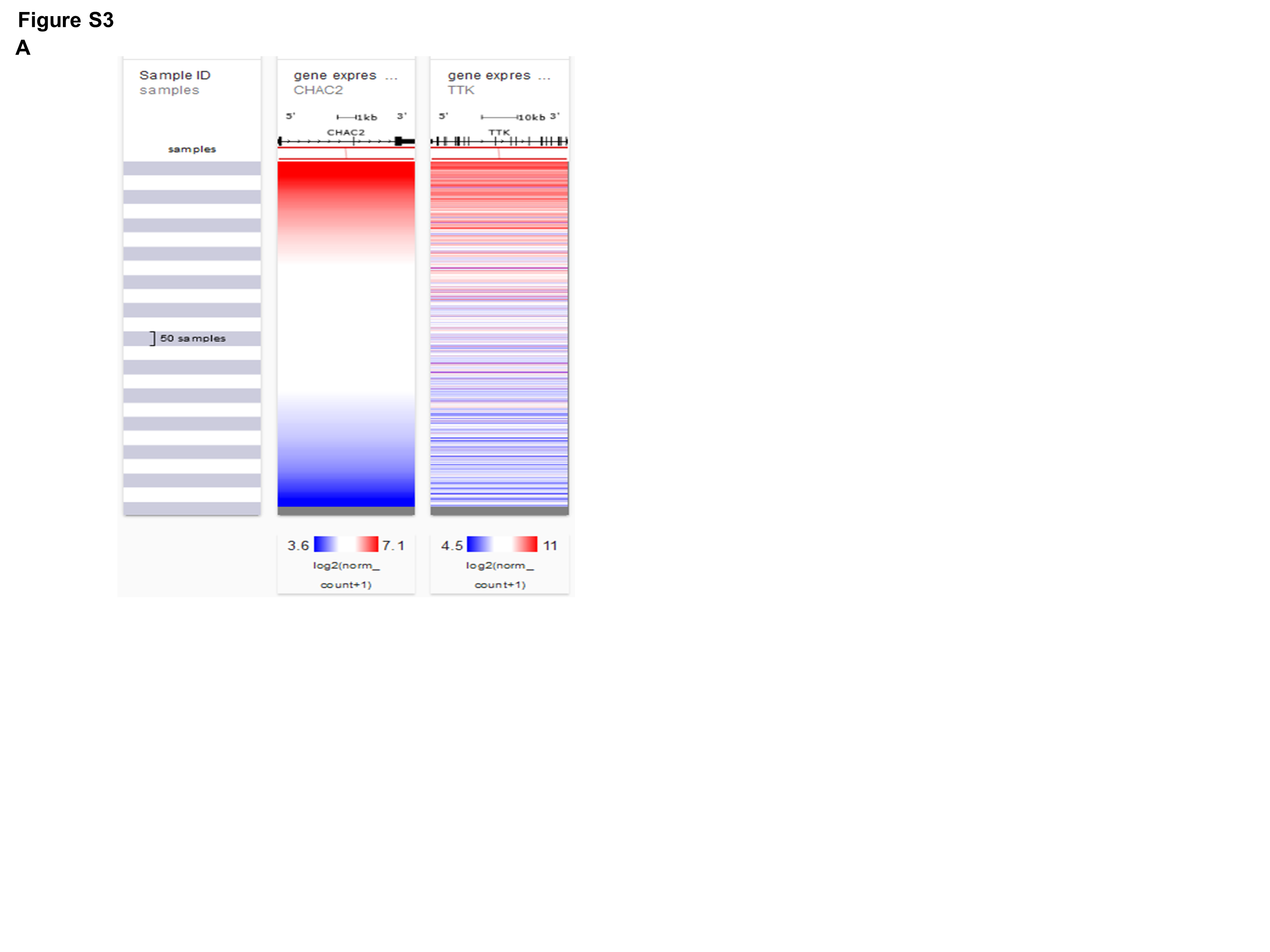

Supplement: Supplementary Figure 5 — Schematic outline of the methodology for cancer informatics analysis of CHAC2 expression. [file Image_5.tif]

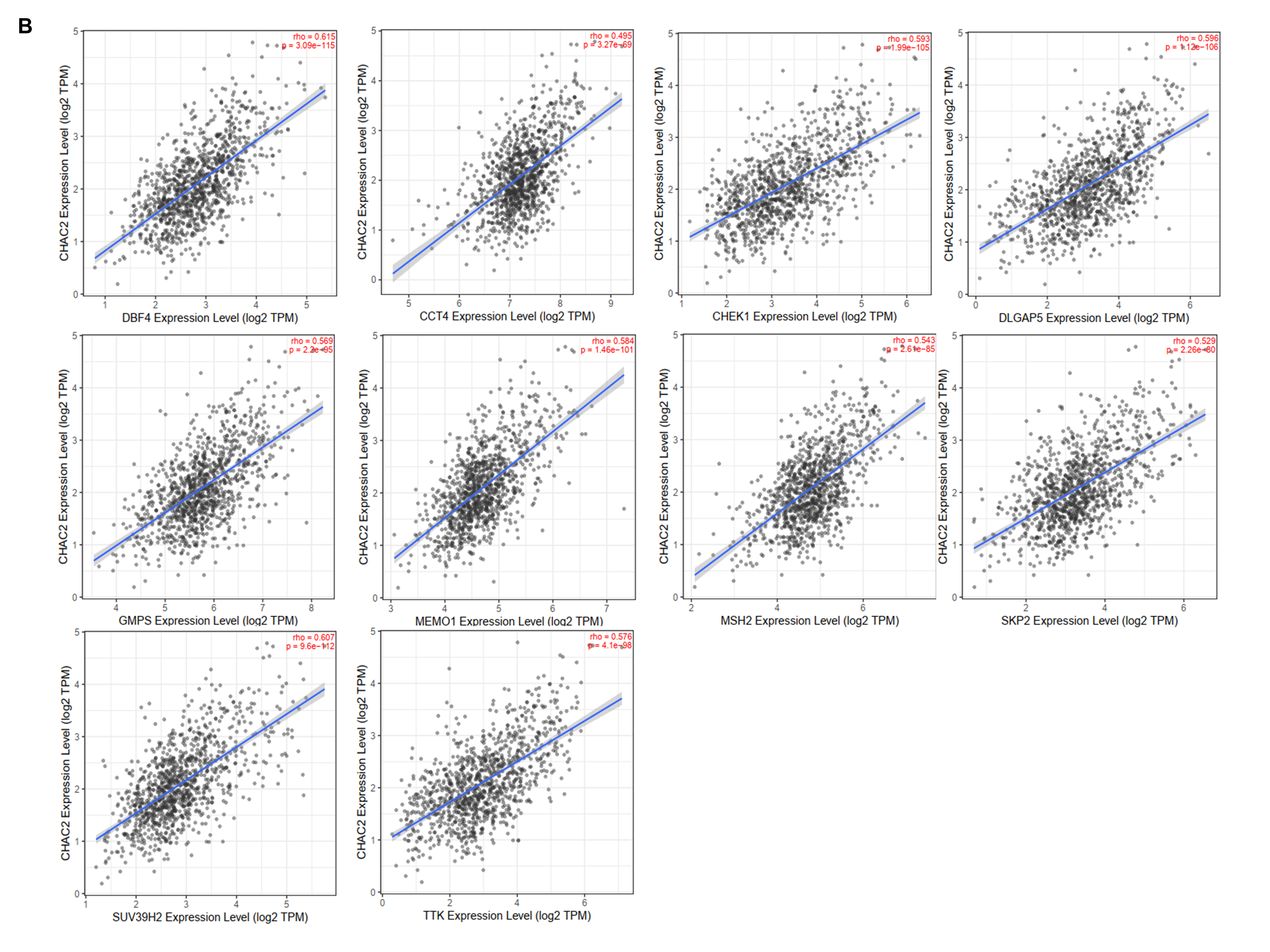

Supplement: Supplementary file 6 [file Image_6.tif]

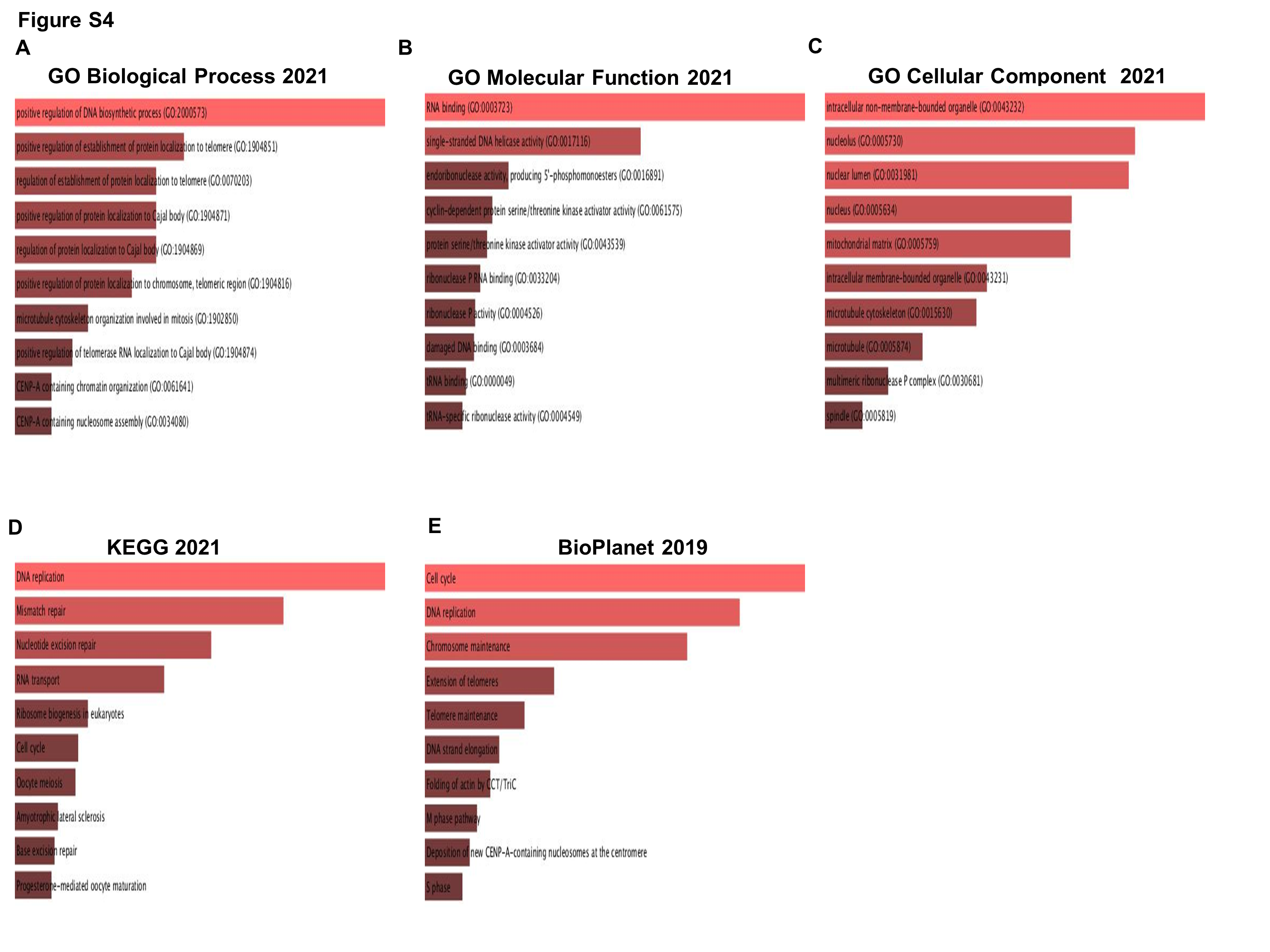

Supplement: Supplementary file 7 [file Image_7.tif]

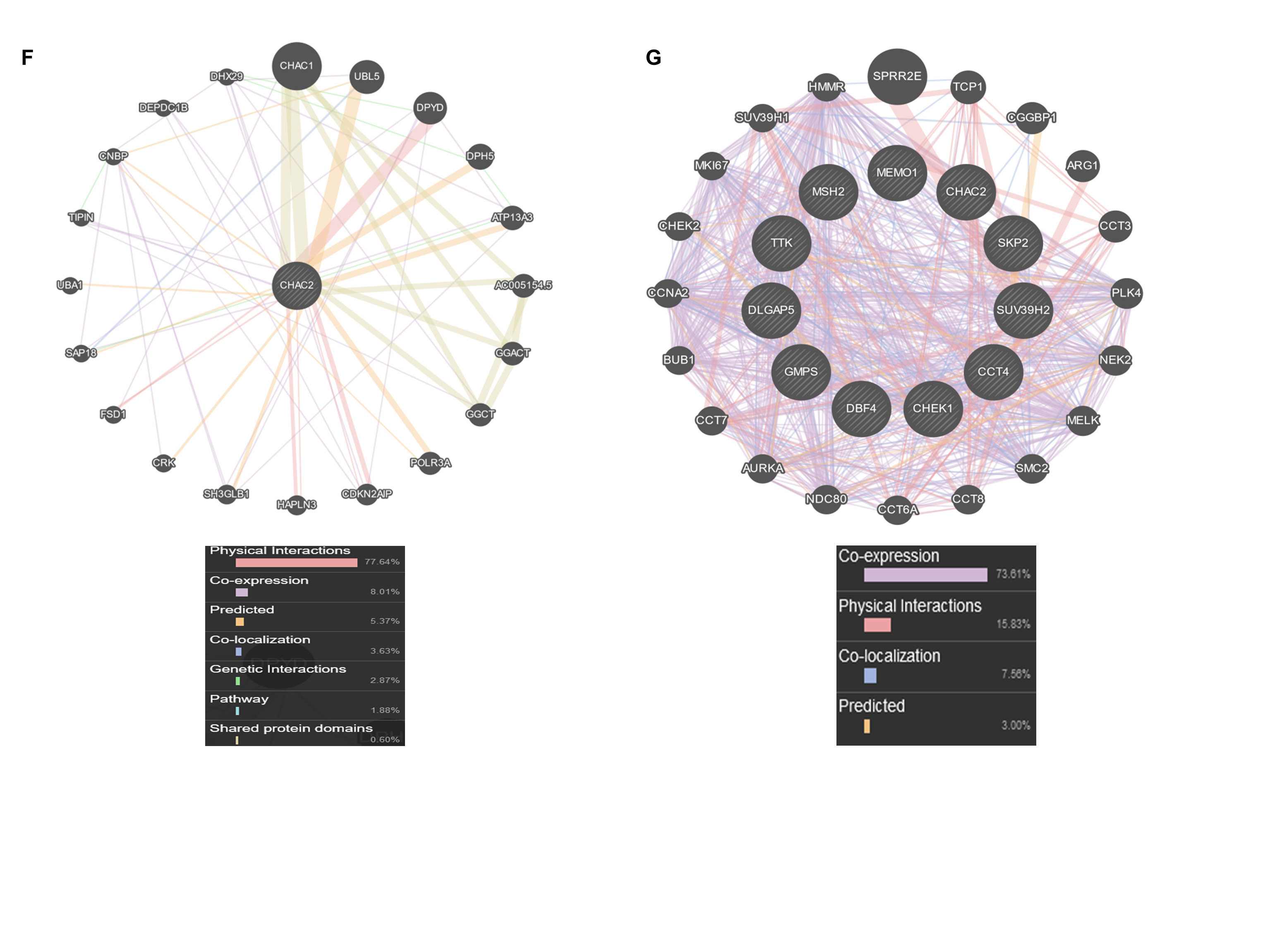

Supplement: Supplementary file 8 [file Image_8.tif]

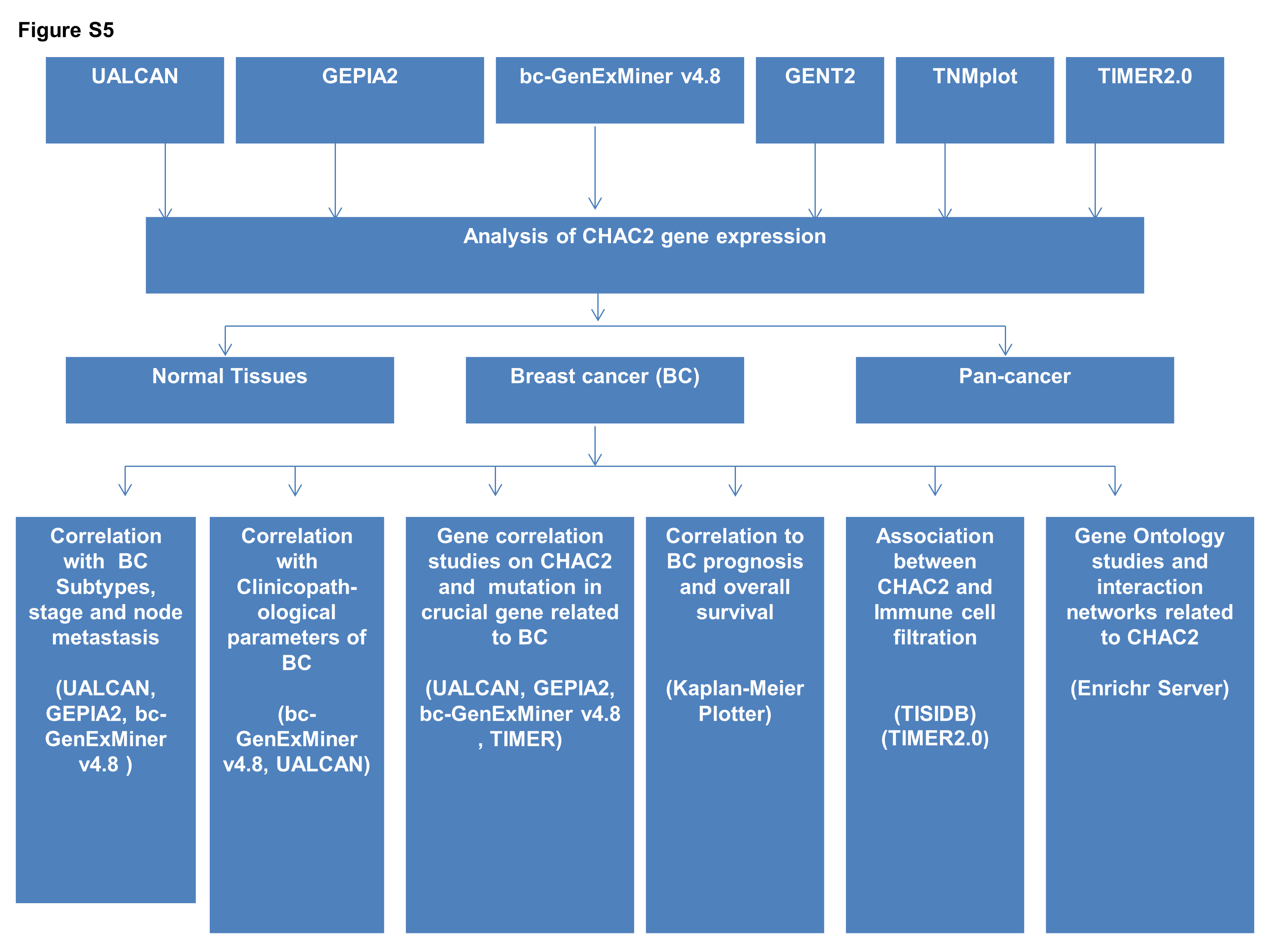

Supplement: Supplementary file 9 [file Image_9.tif]
