# Supplementary material for: Cancer informatics analysis indicates high CHAC2 associated with unfavorable prognosis in breast cancer
Source: Front Oncol. 2022 Dec 9;12:1058931. doi: 10.3389/fonc.2022.1058931 (PMC9780439; doi:10.3389/fonc.2022.1058931)
Supplement: Supplementary file 10 [file Table_1.docx]

**Supplementary Table**

**Table S1** The relationship between CHAC2 mRNA expression and clinicopathological features of breast cancer from UALCAN database.

| **Type of Sample** | **Number of samples** | **Expression of CHAC2** | ***p-*value** |
| --- | --- | --- | --- |
| Normal | 114 | Down-regulated |  |
| Tumor | 1097 | Up-regulated | 1.62E-12 |
| **Individual Cancer Stages** |  |  |  |
| Normal | 114 | Down-regulated |  |
| Stage 1 | 183 | Up-regulated | 9.51E-12 |
| Stage 2 | 615 | Up-regulated | 1.62E-12 |
| Stage 3 | 247 | Up-regulated | 2.23E-12 |
| Stage 4 | 20 | Up-regulated | 1.08E-01 |
| **Patient’s Race** |  |  |  |
| Normal | 114 | Down-regulated |  |
| Caucasian | 748 | Up-regulated | <1E-12 |
| African American | 179 | Up-regulated | 5.56E-12 |
| Asian | 61 | Up-regulated | 3.98E-06 |
| **Patient’s Gender** |  |  |  |
| Normal | 114 | Down-regulated |  |
| Male | 12 | Up-regulated | 3.87E-01 |
| Female | 1075 | Up-regulated | <1E-12 |
| **Patient’s Age (Yrs)** |  |  |  |
| Normal | 114 | Down-regulated |  |
| 21-40 | 97 | Up-regulated | 5.66E-07 |
| 41-60 | 505 | Up-regulated | <1E-12 |
| 61-80 | 431 | Up-regulated | 1.11E-16 |
| 81-100 | 54 | Up-regulated | 2.4E-04 |
| **Major Subclasses** |  |  |  |
| Normal | 114 | Down-regulated |  |
| Luminal | 566 | Up-regulated | 1.11E-16 |
| HER2 positive | 37 | Up-regulated | 3.51E-06 |
| Triple-Negative | 116 | Up-regulated | 1.62E-12 |
| **Menopause Status** |  |  |  |
| Normal | 114 | Down-regulated |  |
| Pre-menopause | 230 | Up-regulated | 6.76E-12 |
| Perimenopause | 37 | Up-regulated | 4.47E-05 |
| Post-menopause | 700 | Up-regulated | <1E-12 |
| **Tumor histology** |  |  |  |
| Normal | 114 | Down-regulated |  |
| IDC | 784 | Up-regulated | <1E-12 |
| ILC | 203 | Up-regulated | 1.76E-03 |
| Mixed | 29 | Up-regulated | 2.2E-04 |
| Other | 45 | Up-regulated | 6.96E-03 |
| Mucinous | 17 | Up-regulated | 2.7E-02 |
| Metaplastic | 9 | Up-regulated | 2.43E-01 |
| INOS | 1 | Up-regulated | N/A |
| Medullary | 6 | Up-regulated | 1.25E-02 |
| **Nodal Metastasis status** |  |  |  |
| Normal | 114 | Down-regulated |  |
| N0 | 516 | Up-regulated | 1.62E-12 |
| N1 | 362 | Up-regulated | <1E-12 |
| N2 | 120 | Up-regulated | 1.49E-08 |
| N3 | 77 | Up-regulated | 4.16E-04 |
| **TP53 mutation status** |  |  |  |
| Normal | 114 | Down-regulated |  |
| TP53-mutant | 334 | Up-regulated | 1.62E-12 |
| TP53-Nonmutant | 698 | Up-regulated | 1.75E-12 |
| **Promoter Methylation Status** |  |  |  |
| Normal | 97 |  |  |
| Tumor | 793 | Hypo-methylated | 1.35E-04 |
